# Internal flow in sessile droplets induced by substrate oscillation: towards enhanced mixing and mass transfer in microfluidic systems

**DOI:** 10.1038/s41378-024-00714-4

**Published:** 2024-06-24

**Authors:** Tianyi Zhang, Peng Zhou, Terrence Simon, Tianhong Cui

**Affiliations:** Department of Mechanical Engineering, University of Minnesota, Minneapolis, MN 55455 USA

**Keywords:** Chemistry, Electrical and electronic engineering

## Abstract

The introduction of flows within sessile droplets is highly effective for many lab-on-a-chip chemical and biomedical applications. However, generating such flows is difficult due to the typically small droplet volumes. Here, we present a simple, non-contact strategy to generate internal flows in sessile droplets for enhancing mixing and mass transport. The flows are driven by actuating a rigid substrate into oscillation with certain amplitude distributions without relying on the resonance of the droplet itself. Substrate oscillation characteristics and corresponding flow patterns are documented herein. Mixing indices and mass transfer coefficients of sessile droplets on the substrate surface are measured using optical and electrochemical methods. They demonstrate complete mixing within the droplets in 1.35 s and increases in mass transfer rates of more than seven times static values. Proof of concept was conducted with experiments of silver nanoparticle synthesis and with heavy metal ion sensing employing the sessile droplet as a microreactor for synthesis and an electrochemical cell for sensing. The degrees of enhancement of synthesis efficiency and detection sensitivity attributed to the internal flows are experimentally documented.

## Introduction

Recently, attention has emerged on microfluidic systems that are based on manipulation of a small sessile droplet on a solid substrate. The sessile droplets with volumes in the nL to μL range are attached to a substrate. They provide an alternative to closed microfluidic systems, such as flow channels, yet offer similar benefits: low sample consumption, high throughput, automation possibilities, and, most importantly, flexibility and versatility^1–3^. Furthermore, the sessile droplet offers additional advantages that include simplicity of fabrication and surface modification, feasibility of operation without a pump, elimination of device failures from channel blockage, and more compliance for unskilled users^4–6^. Sessile droplets on a solid substrate are used in a myriad of microfluidic applications, such as chemical synthesis^7,8^, polymerase chain reaction (PCR)^9,10^, immunoassay^11,12^, electrochemical sensing^13,14^, particle and cell separation and agglomeration^15–17^, and cell culturing^18,19^.

Where applied, flows within sessile droplets are valuable for achieving uniform mixing and rapid mass transport. Without such flows, mixing and mass transfer processes primarily depend on slow molecular diffusion, making microfluidic system procedures time-consuming and inefficient^20–23^. In general, flows can be triggered within a sessile droplet simply by the evaporation of the droplet itself. The heat loss during evaporation generates differences in surface tension along the droplet surface, in which recirculating flows are produced by the Marangoni Effects^24,25^. However, the Marangoni flows in water is uncontrollable to a certain degree, which is extremely sensitive to substances in water, and can be too weak to meet convection needs^26^. Various studies have been reported focusing on enhancing the Marangoni flows by introducing physical or chemical factors, e.g., surfactant, chemical vapor, heating, etc^27–29^. Nevertheless, these factors might not be fully compatible with droplet-based biological or chemical reactors. Usually, generating intense convective flows in small volumes such as sessile droplets is difficult because of low Reynolds numbers^30,31^. As a response, researchers have developed multiple methods to generate internal flows in sessile droplets. Techniques have been developed to transmit different types of external energy into the liquid to actuate fluid motion. For example, electrowetting on dielectrics (EWOD) is used to induce droplet vibration to accelerate mixing by dynamically tuning the wettability of the droplets on a solid surface with an alternating electric field^32,33^. AC electrothermal flow (ACEF) is employed in small-volume samples where an AC electrical field introduces nonuniform Joule heating^34,35^. Surface acoustic waves (SAW) produced on a piezoelectric substrate are applied to transfer momentum to the fluid^36,37^. Magnetic nanoparticles added to the droplet can be magnetically activated to stir the internal fluid with external magnetic fields^38,39^. Rotating disk electrodes (RDE) can be utilized in which a single droplet is confined between a rotating disk and a fixed substrate^40,41^. A droplet-carrying substrate can be driven into vertical or horizontal oscillation causing flows when the droplet vibrates at one of its resonant states^13,42–44^. The above methods have limitations in their applications, however. For instance, the liquid to be actuated in a sessile droplet must be closely linked or in direct contact with the energy delivery components (electrodes for the EWOD and ACEF methods; piezoelectric crystals for SAW; and magnetic nanoparticles and rotating disks for the latter two methods). These requirements put limitations on flexibility and may lead to contamination of liquid samples. The vibrating droplet method is not subject to such limitations, which is attributed to the rigid oscillation of the entire substrate, but such methods may encounter difficulties in selecting the proper driving frequency. This is because the driving frequency must be close to the resonant frequency of the droplet, which is highly sensitive to the droplet volume, droplet composition, and contact interface features^45,46^.

**Fig. 1:**
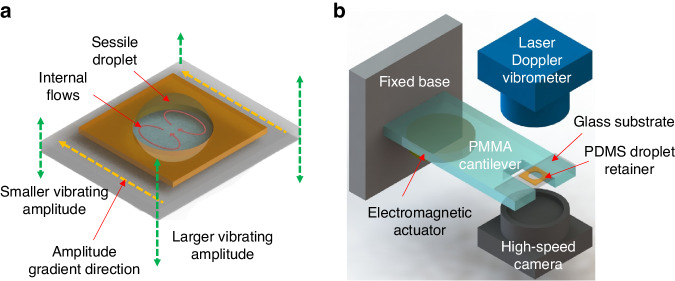
Illustration of oscillating substrate-induced droplet internal flow. **a** Schematic diagram of the substrate oscillating amplitude and the corresponding internal flow field in the sessile droplet. **b** schematic of the experimental setup for the optical vibrational test, particle images velocimetry, and mixing index characterization

In this paper, we propose a novel flow generation strategy to overcome the above-mentioned shortcomings of the vibrating droplet method. The flows within the sessile droplet are generated by vertical oscillation of the substrate with a specific amplitude distribution, which will be controlled by a resonant cantilever beam. The schematic diagram of the substrate oscillating amplitude and the corresponding internal flow field in the sessile droplet are shown in Fig. 1(a). Unlike using the sessile droplet as a liquid resonator, our strategy utilizes a solid resonant structure. The driving frequency in this work is a fixed value determined by mechanical components. In this case, the influence of the droplet resonant frequency on the selection of the driving frequency is eliminated. The oscillation characteristics of the substrate are measured using a laser Doppler vibrometer (LDV). Flow fields inside the sessile droplet are documented with particle image velocimetry (PIV). The generated flows in sessile droplets are employed for a multitude of fluid mechanics applications. The sessile droplet is used successively as a microreactor and an electrochemical cell to study the influence of its internal flows. The mixing index of the droplet micromixer, characterized by dye mixing experiments is evaluated. Silver nanoparticle synthesis tests are conducted to verify the flow-assisted mixing capability of the droplet reactor. In addition, electrochemical methods are used to characterize the effects of flows on mass transfer efficiency using the sessile droplet as a reaction cell. Sensing properties of an electrochemical heavy metal sensor are documented.

## Results and discussion

### Characterization of the oscillating substrate and flow field

**Fig. 2:**
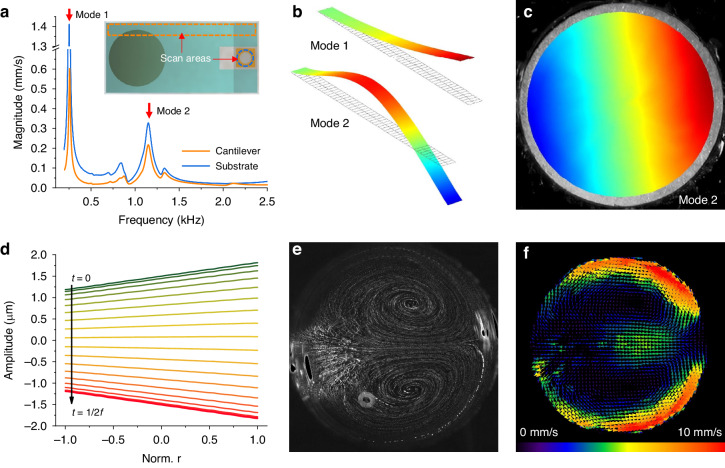
Resonance characteristics of the actuating components and the internal flow characteristics of a sessile droplet. **a** Measured frequency response of the PMMA cantilever and the glass substrate under the retainer, where two vibration modes are labeled with red arrows. Insert in (**a**) is a figure indicating the scan areas of the laser Doppler vibrometer. Plots (**b**) and (**c**) are mode shapes of the cantilever and the glass substrate in which the color ranging from blue to red represents vibrating velocity from −1.4 mm/s to 1.4 mm/s for mode 1 in (**b**), −0.5 mm/s to 0.5 mm/s for the mode 2 in (**b**), and 0.25 mm/s to 0.4 mm/s in (**c**). Plot (**d**) is the measured vibrating amplitude distributions along the diameter of the droplet retainer during half a period of oscillating (green to red indicates different time steps, with a time interval between two adjacent curves of 1/41,400 s) at a driving frequency of 1.15 kHz. Plot (**e**) is the flow pattern image of the 60 μL sessile droplet measured with the same driving frequency and voltage as in (**d**). Plot (**f**) is the distribution of velocity vectors of the internal flow field obtained from the particle tracking data. The voltage *V*_*ac*_ = 0.1 V in the vibrational measurements of figures (**a**), (**b**), and (**c**) and *V*_*ac*_ = 0.05 V in the test of (**d**), (**e**), and (**f**). A full video of (**e**) is available in the supplementary material (Video S1)

The frequency response of a Poly (methyl methacrylate) (PMMA) cantilever beam and a glass substrate is obtained from the LDV using the experimental setup shown in Fig. 1b. The electromagnetic actuator is driven with a frequency chirp signal (from 0.1 to 2.5 kHz). Meanwhile, the out-of-plane vibration signal is Fourier transformed. By averaging the results of all the scan points in the scan areas shown as the insert of Fig. 2a, the frequency responses of the cantilever and the substrate are obtained. They are plotted in Fig. 2a. Two clear resonance peaks (at 0.26 kHz and 1.15 kHz) are observed in both curves, corresponding to the first two orders of the vibration modes of the cantilever beam. According to our experiments, the effect of the tiny water droplet on the frequency response of the cantilever can be neglected. Subsequently, the 3D mode shape patterns of the cantilever are calculated based on the phase information of all the scanned points, as shown in Fig. 2b. The color scale from blue to red indicates increasing vibrating velocities. Both the first- and second-order modes produce vertical velocities with gradients at the free end of the cantilever beam. In all subsequent experiments, the cantilever is driven into the second-order mode (1.15 kHz). A lower excitation frequency (0.26 kHz) is avoided since it is closer to the resonant frequencies of the first few orders of the droplet where liquid can spill due to violent deformation^42^. The vibrating velocity distribution of the glass substrate within the circular retainer area is shown in Fig. 2c. A velocity distribution with a gradient inward from the free end of the cantilever beam is shown. It is worthy of mention that the two plots in Fig. 2b and the pattern in Fig. 2c have different color scales to make the images clearer (the scales are indicated in the caption of Fig. 2). In addition, vibrating amplitude distributions of the substrate along the diameter of the droplet retainer within half an oscillating period are shown in Fig. 2d, where the curves from green to red indicate the different time steps within half a cycle (the time interval between two adjacent curves is 1/41400 s). With a driving frequency of 1.15 kHz and voltage of 0.05 V, the vibrating amplitude of the substrate is ±1.19 μm on the anchor side and ±1.81 μm on the free end side, showing rigid oscillation with an amplitude gradient along the cantilever beam.

Subsequently, the internal flow field within a sessile droplet induced by substrate oscillation with such an amplitude gradient is characterized using PIV method. The cantilever vibration is driven using the same parameters shown in Fig. 2d. Meanwhile, images of fluorescent particles 2 μm in diameter excited by a blue LED are captured with the high-speed camera. Trajectories representing the particles’ movements are obtained by comparing the changes of the images over 20 consecutive frames taken in 0.1 s (with a frame rate of 200 per second) using the ImageJ software. The results are shown in Fig. 2e. Two symmetrical vortices are visible in the flow pattern. Each occupies half the space of the droplet. They converge at the midline. A full video of Fig. 2e is available in the supplementary material (Video S1). The vector image of the internal flow field is obtained also by using the ImageJ software, as plotted in Fig. 2f. The flow direction at the midline is aligned with the vibrating amplitude gradient of substrate oscillation, from higher to lower amplitudes. Furthermore, to demonstrate reproducibility, the correlations of vibrating amplitude distribution with flow patterns are experimentally described using a different experimental setup: the width of the glass slide is lengthened to 25 mm while the retainer is attached to different locations on the slide (indicated in Fig. S1). The vibrating amplitude distribution within the retainer when set at various locations can be completely different due to the existence of a nodal line (zero amplitude) near the tip of the cantilever beam (Fig. 2b). The results are shown in Fig. S1, which reveals the correspondence between the vibrating amplitude distributions of the droplet substrate and the flow patterns in the droplet. In addition, flow trajectories are recorded using various driving voltages with the original experimental setup. The results are shown in Fig. S2. As the voltage increases, the vibrating velocity of the glass substrate increases, and the particle trajectories show increased flow speeds. Shown is that with this proposed method, the flow characteristics within the sessile droplet are completely controlled by the amplitude distribution of the oscillating substrate, rather than the cantilever vibration or droplet vibration, and the proper driving frequency is only relevant to the mechanical structure of the actuating components. Such a flow generation strategy physically isolates the sessile droplets from the energy delivery components, allowing for completely non-contact operation. Moreover, without a need to worry about the effect of the droplet on the selection of the driving frequency, our approach provides a clear advantage over the methods that are dependent on droplet resonance. Since the interior of the droplet is filled by two symmetrical vortices, this internal flow generation strategy can be used to accelerate mixing of reactants and the movement of molecules toward the bottom where the sensing surface resides. In the following two sections, mixing indices and mass transfer coefficients in a sessile droplet are evaluated under internal flows.

### Characterization of mixing index and silver nanoparticle synthesis

**Fig. 3:**
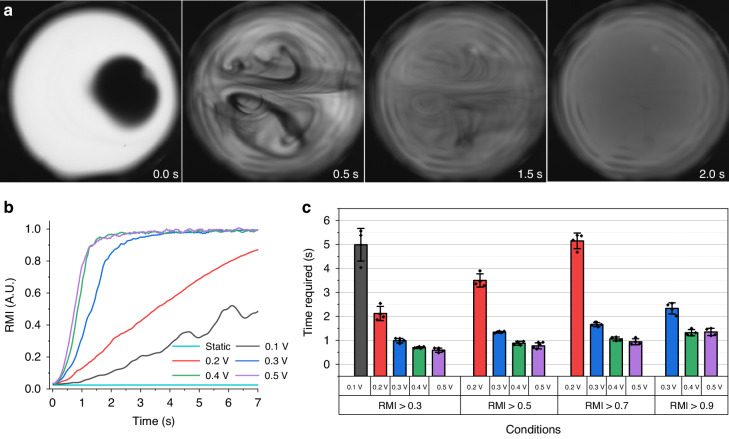
Characterization of the mixing index using a sessile droplet as a mixer. **a** shows optical images of the sessile droplet in the retainer at different time steps in the mixing process. The substrate is actuated into oscillation at a driving frequency of 1.15 kHz and a voltage of 0.3 V during the whole process. Plot (**b**) is the calculated relative mixing index (RMI) as a function of the mixer’s working time, under various driving voltages of the actuator. Plot (**c**) is the working time required to reach a certain RMI value for the different driving voltages. The bars show standard deviations over four independent measurements. In the above experiment, 1 μL methylene blue dye (1 mmol/L) is injected into the sessile droplet containing 59 μL deionized water. A full video of (**a**) is available in the supplementary material (Video S2)

Mixing performance by internal flows is characterized using a dye mixing process. A dye of 1 μL methylene blue (1 mmol/L) is injected into the sessile droplet containing 59 μL deionized water and the glass substrate is driven into oscillation immediately afterward. The images showing mixing over time are recorded by a high-speed camera. Images with a driving frequency of 1.15 kHz and a voltage of 0.3 V at specific time steps are shown in Fig. 3a. In the initial stage, there is a clear boundary between the injected dye and the water. The boundary is soon broken by symmetric vortices once oscillation of the substrate is initiated. After 2 s of oscillation, the methylene blue dye is almost uniformly distributed throughout the entire droplet. A full video of Fig. 3a is available in the supplementary material (Video S2). The relative mixing index (RMI) as a function of time is calculated using the fully mixed solution as a reference to quantitatively evaluate mixing capabilities under various driving voltages. The results over 7 s are shown in Fig. 3b. According to the equation in “Test of mixing index and silver nanoparticle synthesis”, RMI values ranging from 0 to 1 indicate unmixed to fully mixed. For the static case, there is almost no mixing over 7 s. The RMI increases faster as the driving voltage is increased, demonstrating that a larger oscillation amplitude of the substrate leads to higher mixing efficiency. Mixing times required to reach certain RMI values for different driving voltages are shown in Fig. 3c. Standard deviations over four independent measurements are reflected by the error bars. For the static droplet, which is not shown in this figure, a time of 415 s is required to reach an RMI of 0.3. The experimental results for mixing under static conditions are in Fig. S3. With the introduction of substrate oscillation, this time is 5.0, 2.1, 0.99, 0.70, and 0.59 s using driving voltages from 0.1 to 0.5 V. These mixing times are significantly faster than in the static case. An RMI of 0.9 represents an almost fully mixed process. The time to reach this value is as short as 1.35 s in the test with a driving voltage of 0.5 V. Thus, the internal flow generation strategy proposed in this paper enables the sessile droplet to become a highly efficient micromixer that could be applied to a droplet-based chemical microreactor.

**Fig. 4:**
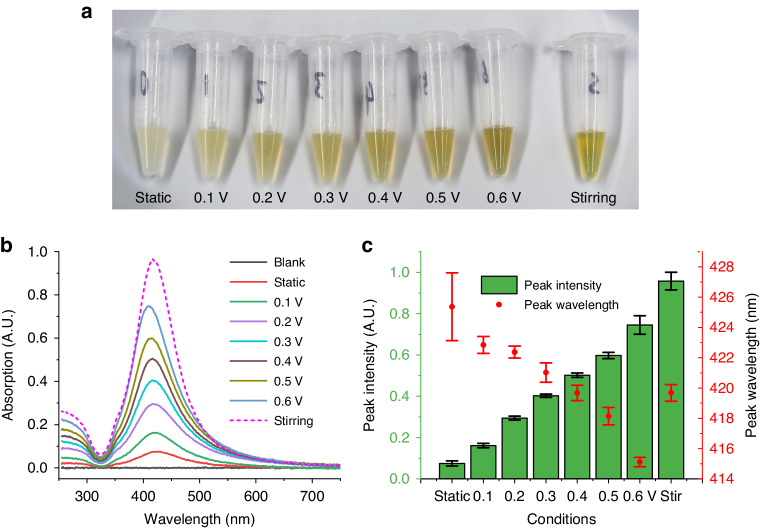
Experimental results of silver nanoparticle synthesis using sessile droplets as reactors. **a** is an optical image of the synthesized silver nanoparticle solutions using different driving conditions, (**b**) is the absorption spectrum of the silver nanoparticle solutions, (**c**) is the peak intensity and peak wavelength obtained from figure (**b**) under various driving conditions. Bars indicate standard deviations obtained with four independent synthesized samples. For the 60 μL sessile droplet reactor, 6 μL of silver nitrate solution (10 mmol/L) is added into a 54 μL hydroxylamine hydrochloride solution (1.67 mmol/L) containing sodium hydroxide (3 mmol/L) when the driving frequency is 1.15 kHz. For the stirring conditions, the volume of two reactants is 60 and 540 μL, respectively, at a stirring speed of 1600 rpm

For proof of concept, the sessile droplet with substrate oscillation is employed as a synthesis unit to control the generation of silver nanoparticles. A 54 μL HONH_2_·HCl solution (1.67 mmol/L) is added first to form the droplet, then oscillation of the substrate is initiated before adding a 6 μL AgNO_3_ solution (10 mmol/L). After the reaction is complete, the mixture is collected in centrifuge tubes, as shown in Fig. 4a, together with the sample synthesized in a 1.5 ml centrifuge tube (total reaction volume of 600 μL) using a magnetic stirrer (1600 rpm). As the driving voltage is gradually increased from 0 (static) to 0.6 V, the color of the mixture changes from gray to yellow. The gray color shows the cross-linked large silver particles, while the unaggregated silver nanoparticles show a yellow color in the solution. With the increase of substrate oscillating intensity, the concentrations of silver nanoparticles also gradually increase. Quantitative characterization of silver nanoparticle solutions is accomplished by absorption spectroscopy measurements. The results are in Fig. 4b, as relative absorption using the solution without silver nanoparticles as a reference. Single absorption peaks at a wavelength near 420 nm can be observed in each result, indicating the generation of silver nanoparticles. The peak height and wavelengths are then extracted, and their values for different driving conditions are plotted in Fig. 4c. Bars are determined using four independent mixtures with the same driving conditions. As the driving voltage of the substrate increases, absorption peak intensities rise and wavelengths reduce, indicating higher nanoparticle concentrations and smaller particle sizes. The absorbance at the absorption peak under the static condition, the condition of oscillation under 0.6 V, and with stirring are 1.19, 5.56, and 9.08 times that of the reference sample (without silver nanoparticles), respectively. Particles synthesized with a magnetic stirrer show an even higher concentration, but with bigger particle sizes compared with those of droplets driven with 0.6 V. This may be caused by regions with different flow rates in the droplets. Although the substrate oscillation method used in this work may not offer the excellent mixing performance of a magnetic stirrer handling a large volume (600 μL), it shows significant advantages with non-contact handling methods of samples in small droplets (60 μL).

### Electrochemical tests of mass transfer coefficients and metal ion detection

**Fig. 5:**
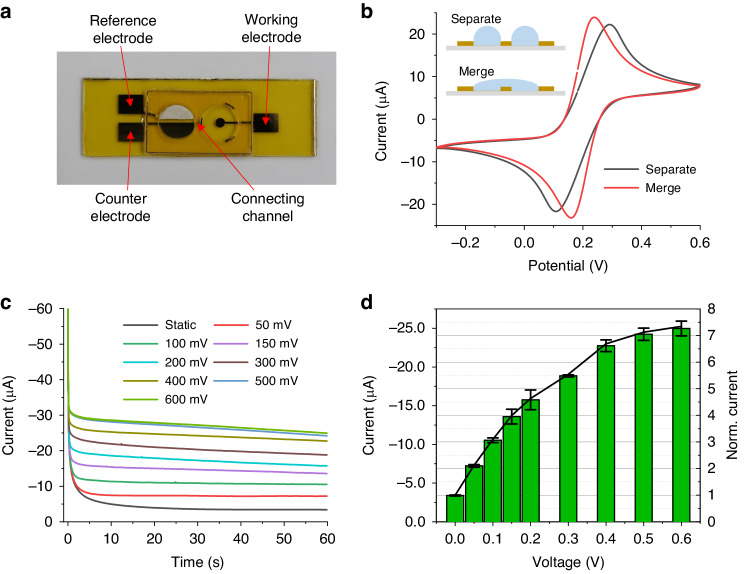
Characterization of mass transfer coefficients using sessile droplets as a sample for an electrochemical sensor. **a** is the electrochemical sensor with three electrodes for the chronoamperometry test, **b** is a comparison of the cyclic voltammetry curves using separated and merged sample droplets, **c** are i-t measurement results of a 60 μL sessile droplet sample at various driving voltages of the actuator. The driving frequency is 1.15 kHz. The plot (**d**) is the averaged current of the last second in (**c**) as a function of the driving voltage of the actuator. Bars indicate standard deviations obtained with four independent measurements. The normalization used the static current as a reference. The droplets contain 10 mmol/L [Fe (CN)_6_]^3−^/ [Fe (CN)_6_]^4−^ (1:1) and 0.5 mol/L KCl. The potential of the working electrode is −0.6 V vs. the reference electrode for the i-t measurements

Mass transfer properties under internal flows are characterized using the chronoamperometry method. A partially separated three-electrode system, as shown in Fig. 5a, is used for the tests. The connectivity of the working electrode to the counter and reference electrodes is verified by comparing cyclic voltammetry (CV) scans between separated and merged conditions, as shown in Fig. 5b. The CV curve obtained using two individual droplets shows a slight increase in the difference between the two peak potentials, indicating full connectivity in the narrow channel. During calibration of mass transport on the working electrode, [Fe (CN)_6_]^3−^ is reduced to [Fe (CN)_6_]^4−^ by a lower potential, and current from the reaction reflects a mass transfer of ferricyanide ions^47^. The cantilever beam is driven into the second-order mode and a 1-min-long time record of the current is taken. Evaporation of the droplet is negligible during this short-time test. The current–time plot for different substrate oscillating conditions is shown in Fig. 5c. In the absence of oscillation, the current shows a gradual downward trend, indicating a growth of the diffusion layer over time. The current proceeds nearly to steady state at the final stage of the test (50–60 s), which indicates a stabilized diffusion layer. In this region, the mass transfer coefficient tends to be stabilized and is mainly dominated by natural convection^48^. By actuating the substrate into oscillation, the reduction current increases according to the driving voltage. The growth of the current is attributed to convective mass transport induced by the internal flows. Note that the current cannot be stabilized when operating at higher drive voltages, which may be induced by reduced concentrations of the reactants ([Fe (CN)_6_]^3−^) due to overall consumption over the entire volume of the droplet. The average current at the last second (59–60 s) of each measurement is calculated and normalized using the static case as a reference. The results are shown in Fig. 5d. The bars indicate standard deviations computed from four independent tests. The solutions in both retainers are replaced with fresh ones before each measurement. The maximum mass transfer rate obtained by driving the substrate with 0.6 V is 7.3 times that in the static droplet. An enhanced mass transfer coefficient is expected to improve efficiency for the electrochemical sensing processes when using droplet samples.

**Fig. 6:**
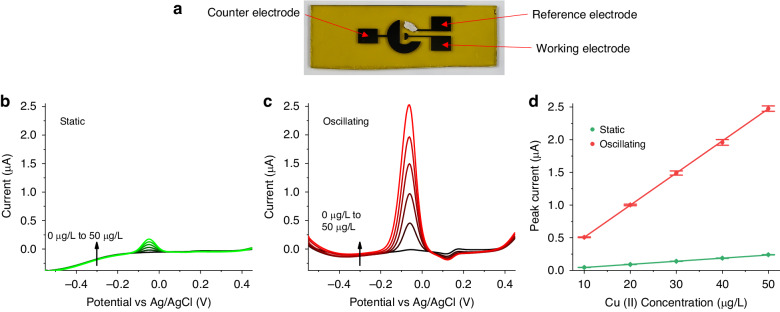
Electrochemical detection of heavy metal ions using a sessile droplet sample. **a** shows the three electrodes for the anodic stripping voltammetry analysis (before attaching the droplet retainer), (**b**) and (**c**) are anodic stripping voltammetry results for Cu^2+^ sensing in a 60 μL droplet simple without and with substrate oscillation, and (**d**) are the calibration plots of Cu^2+^ in static and oscillating conditions. The bars are standard deviations from four independent measurements. The driving frequency and voltage are 1.15 kHz and 0.6 V, respectively. The analyte solution is 0.1 mol/L sodium acetate buffer solution (pH 4.5) that contains various concentrations of Cu^2+^

Subsequently, the anodic stripping voltammetry (ASV) method is utilized for the detection of copper ions in a 60 μL sessile droplet sample as an electrochemical cell. The three-electrode system shown in Fig. 6a is applied. Sensing performance changes induced by substrate oscillation are evaluated. In an ASV test of heavy metal ions, the anodic peak current during stripping is used to quantify the metal deposited on the working electrode. The cantilever beam is driven into vibration with a frequency of 1.15 kHz during the metal reduction step. The stripping current curves are calculated by subtracting background scanning from analytical scanning. The curves are plotted in Fig. 6b, c, corresponding to the static and substrate oscillation conditions, respectively. As Cu^2+^ concentration increases, the stripping peak current gradually increases in both static and oscillation conditions, which is due to more copper being oxidized during stripping. The peak current with substrate oscillation is much higher compared with the static operation peaks due to more rapid mass transport. Calibration of the peak current as a function of copper ion concentration is shown in Fig. 6d. The bars are computed as standard deviation from four repeated tests, with a new sample droplet in each test. The results show linear fits with adjusted R-Squared fitting values of 0.9991 and 0.9999 in the static and oscillating cases, respectively, demonstrating linearity for both conditions. The sensitivities in two conditions are 4.76 nA/μg·L^−1^ and 49.13 nA/μg·L^−1^, demonstrating enhancement ratios of 10.3 when substrate oscillation is introduced. Since this convective mass transfer strategy utilizes rigid movement of the sample substrate, it is also suitable for application of electrode arrays and multi-sample tests.

## Conclusion

Here, we propose a simple, non-contact strategy to generate internal flows in sessile liquid droplets for multiple applications. This strategy is achieved by driving a rigid substrate that supports the droplet into oscillation with certain amplitude distributions. The driving frequency of this approach is independent of the droplet and can be determined by the mechanical structure. With the flow-induced improvement in mixing and mass transport capabilities, performance of the sessile droplet as a microreactor, as well as an electrochemical sensor is experimentally documented in this work.

The substrate’s oscillation with a prescribed amplitude gradient is provided by a cantilever beam that is excited to give a second-order vibration mode. Particle image velocimetry tests indicate that symmetric vortex flows within the droplet are produced once the substrate is driven into oscillation with such gradient. The flow pattern and direction are related to the vibrating amplitude distribution of the substrate surface. The circular flows can be employed to modify mixing efficiency and mass transfer coefficients within the droplets. High-speed video of dye mixing indicates that oscillation-induced flows significantly improve the mixing index of the droplet micromixer. Higher concentrations and smaller sizes of silver nanoparticles are synthesized in the droplet reactor, attributed to better mixing performance, as shown by the absorption spectroscopy measurements. The electrochemical measurements indicate that the mass transfer rate from the droplet solution to the bottom electrode is improved by a factor of over seven through substrate oscillation. Enhanced sensitivity is achieved with such flows in metal ion detection using the droplet as an electrochemical cell. To summarize, the internal flow generation strategy achieved by actuating the substrate into oscillation with certain amplitude distributions shows excellent promise in handling sessile droplets, especially when higher efficiency mixing and rapid mass transport are sought.

## Methods

### Reagents

The reagents in our experiments are latex beads (fluorescent green), methylene blue, silver nitrate, sodium hydroxide, hydroxylamine hydrochloride, potassium chloride, potassium hexacyanoferrate (III), potassium hexacyanoferrate (II), acetic acid, sodium acetate, and copper solution (1 g/L), chloroplatinic acid, from Sigma-Aldrich (St. Louis, MO, United States); 98% sulfuric acid from Fisher Scientific (Hampton, NH, United States). Solutions in the test are made with deionized water from AmeriWater silex deionization system (Dayton, OH, United States).

### Device fabrication

A polymer-based cantilever beam structure is used to produce an oscillation with certain amplitude distributions on a glass substrate. The PMMA cantilever beam (93 mm long, 49 mm wide, and 6 mm thick) is fabricated with a laser cutter. A glass slide (37.5 mm long, 12.5 mm wide, and 0.5 mm thick) is attached at the tip of the cantilever beam, acting as the substrate to carry the droplets. A hollow area is designed at the tip of the cantilever beam to allow observation of the droplets on the glass slide. A rectangular polydimethylsiloxane (PDMS) pad 0.5 mm thick is used as the droplet retainer to hold the droplets in place. The PDMS retainer is fabricated following these steps: precursor and curing agent are mixed at a 10:1 weight ratio, degassed under vacuum, and cast into a rectangular PMMA mold with a thickness of 0.5 mm. The excess uncured PDMS is removed with a razor blade to ensure a flat surface. The bottom layer of the mold is a double-sided polyimide tape for attachment with the substrate. Then a circular hole 6 mm in diameter on the PDMS pad is made with a biopsy punch.

A three-electrode system (working, counter, and reference electrodes) is fabricated for mass transfer calibration and heavy metal ion sensing. Electrodes made of gold are patterned on a polyimide (PI) sheet bonded to the glass slide. The gold electrode pattern is fabricated using the following steps: The PI sheet with the glass slide is first attached to a shadow mask made of polyester. Gold electrodes (100 nm thick) on a PI sheet are formed by sputtering. Afterwards, the reference electrode area is covered with Ag/AgCl paste, followed by high-temperature curing (160 °C for 45 min). The fabricated electrode for the mass transfer test is shown in Fig. 5a. The working electrode has a diameter of 1.5 mm (area 2.22 mm^2^), while the area of the counter electrode is much bigger (11.88 mm^2^) to weaken polarization on the electrode^49^. During the detection of metal ions, a reduced area of working electrode (1.34 mm^2^) is used for the same reason, as shown in Fig. 6a. In addition, a counter electrode (area 12.77 mm^2^) is plated with platinum (10 mmol/L H_2_PtCl_6_ in 0.5 mol/L sulfuric acid, 0 V vs. reference electrode for 0.5 min.) to protect the counter electrode from oxidation.

### Resonant response and flow field characterization

Figure 1b shows the experimental settings for characterization of the vibrational properties and flow field. A laser Doppler vibrometer (PSV-400 Polytec Inc., Baden-Württemberg, Germany) is employed to detect the vibrating amplitude of the cantilever beam and the glass substrate. The excitation voltage *V*_*ac*_ is enlarged with a power amplifier, then used to actuate an electromagnetic actuator (EX 45S, VISATON GmbH & Co. KG, Haan, Germany) fixed on the cantilever beam. For the vibration measurement of the cantilever or the substrate, laser light is focused on the upper surface. The out-of-plane vibrating velocity of the solid surface is then measured.

The flow field in a sessile droplet is characterized by using PIV with a blue LED light source and fluorescent particles (green). The droplets are composed of green fluorescent spheres diluted 10,000 times. The blue LED illuminates the droplet from the oblique upper side. The excited fluorescence from the particles passes through an optical filter placed under the droplet retainer and is recorded using a high-speed camera placed underneath (GS3-U3-23S6M-C, FLIR Systems, Wilsonville, OR, United States). The internal flow trajectories in the droplet are obtained by comparing particle locations in different photo frames.

### Test of mixing index and silver nanoparticle synthesis

The mixing index within the droplet mixer is characterized by observing the mixing process of 1 μL methylene blue dye (1 mmol/L) and 59 μL deionized water. A red LED light source and a soft light diffuser are arranged directly above the droplet retainer. The red light is chosen since methylene blue presents an absorption peak wavelength near 665 nm^50^. Oscillation of the substrate is turned on immediately after the dye is injected into the droplet with a pipette gun. The high-speed camera below the droplet retainer is used to record the process of mixing over time. The pixel grayscale value of the retainer images is extracted using ImageJ software (rsb.info.nih.gov/ij/) to calculate the relative mixing index (RMI) as a function of time^51,52^. The RMI is calculated using the following equation:

$${RMI}=\sqrt{\frac{1}{N}\mathop{\sum }\limits_{i=1}^{N}{({c}_{{fi}}-{\overline{{c}_{f}}})}^{2}\,}\Bigg/\sqrt{\frac{1}{N}\mathop{\sum }\limits_{i=1}^{N}{({c}_{i}-{\overline{{c}_{f}}})}^{2}\,}$$

where *c*_*i*_ is the intensity of pixel *i* during the mixing procedure, and *c*_*fi*_ is the intensity of pixel *i* after the droplet is fully mixed. The averaged intensity over all the *N* pixels in the region of interest after full mixing is $\bar{{c}_{f}}$. The selection region is a concentric circle with a smaller size than that of the retainer, thus excluding false signals due to droplet edge deformation under actuation. After each mixing process, the oscillation is adjusted to high amplitude to fully mix the droplet, then the pixel intensity *c*_*fi*_ is measured using the same amplitude as the one used in the mixing process.

Silver nanoparticles are synthesized in the sessile droplet by adding 6 μL AgNO_3_ solution (10 mmol/L) into 54 μL HONH_2_·HCl solution (1.67 mmol/L) containing NaOH (3 mmol/L)^53^. After HONH_2_·HCl solution is added, the substrate is driven into oscillation (excluding static cases) before the addition of the AgNO_3_ solution. For static cases, the reaction solution lies idle for 5 min to ensure that the reaction is as complete as possible. For oscillating cases, the substrate is driven into oscillation for 1 min, followed by standing idle for 4 min. To reduce the effects of evaporation and light on droplet contents, the experimental setup was placed in a dark humidity box during synthesis. The concentration and particle size of the synthesized silver nanoparticles are determined by the optical absorption technique. A higher concentration of nanoparticles absorbs more light, leading to a higher absorption rate. In addition, as the particle size increases, the absorption peak wavelength shifts from lower to higher wavelengths^54^. To achieve the measurement, 200 μL solution (from the mixture of reaction solutions for four replicate experiments, 50 μL in each) containing particles is diluted to 1 mL and centrifuged. A dilution factor of 5 is applied since a high concentration would exceed the upper limit of the measuring range. To avoid blockage of light by cross-linked large silver particles, a centrifuge step is applied using 1500 rpm for 20 min before the sample absorption test to remove the cross-linked large particles and retain the nanoparticles. Then 0.75 mL of the upper liquid of the centrifuged solution is transferred into a fluorescence micro-cuvette with an optical path length of 10 mm. Then a broadband light source (LDLS, Energetiq Technology Inc., Woburn, MA, United States) is focused onto the cuvette. Absorption of light of about 420 nm wavelength is measured with a spectrometer (AvaSpec-ULS2048CL-EVO, Avantes B.V., Apeldoorn, The Netherlands).

### Test of mass transfer coefficients and heavy metal ion sensing

An electrochemical workstation (PGSTAT302N Metrohm, Riverview, FL, United States) is used to measure the mass transfer rates as well as sensing the properties of heavy metal ions with chronoamperometry and anodic stripping voltammetry.

The three-electrode sensor fabricated on PI film shown in Fig. 5a is used for the mass transfer measurement. The working electrode is separated from the counter and reference electrodes with a dual-retainer setup. The electrodes are connected through a narrow channel between the two retainers. Reduction on the working electrode brings polarization of the counter electrode, in which the reduction product ([Fe (CN)_6_]^4^^−^) is transferred into [Fe (CN)_6_]^3^^−^ by a high potential. If the electrodes were in the same space, the counter and working electrodes form a generator-collector system^55^ in which the concentration of the analyte ([Fe (CN)_6_]^3−^) near the working electrode is disturbed. A cyclic voltammetry test is used first to check the connectivity of the channel by comparing CV curves in the separated and merged conditions^56^. The CV is conducted using a 10 mmol/L K_3_[Fe (CN)_6_]/K_4_[Fe (CN)_6_] (1:1) in 0.5 mol/L KCl solution, with a scan window of −0.3 V to 0.6 V vs. Ag/AgCl reference electrode, and a scan rate of 0.1 V/s. The chronoamperometry test is then conducted for the mass transfer test using the same solution. The applied potential for the test is −0.4 V vs. the reference electrode. The reduction current is recorded while substrate oscillation is maintained at various amplitudes. After each test, the solution is removed, and the retainer is refilled with 60 μL of the new solution.

Sensing of copper ions is achieved using the ASV method with the electrodes shown in Fig. 6a. Detection of different concentrations of Cu^2+^ in a 0.1 mol/L sodium acetate buffer solution (pH 4.5) is conducted within a 60 μL droplet. The detection process consists of five steps: preconditioning of working electrode, background scanning, metal reduction, analytical scanning, and postconditioning of the working electrode. Two conditioning steps have a working potential of 0.45 V vs. the reference electrode and a working time of 60 s. In the two scanning steps, square wave voltammetry is used with a scan window of −0.55 V to 0.45 V, an amplitude of 25 mV, a frequency of 30 Hz, and steps in potential of 5 mV. In the 60 s metal reduction step, the working potential is −0.55 V vs. the reference electrode. The glass substrate is driven into oscillation only during metal reduction. The analyte solution is changed to fresh solution after each test.

### Supplementary information

Supplementary information

Supplementary video 1

Supplementary video 2
