# Supplementary material for: Internal flow in sessile droplets induced by substrate oscillation: towards enhanced mixing and mass transfer in microfluidic systems
Source: Microsyst Nanoeng. 2024 Jun 24;10:86. doi: 10.1038/s41378-024-00714-4 (PMC11196738; doi:10.1038/s41378-024-00714-4)
Supplement: Supplementary file 1 — Supplementary information [file 41378_2024_714_MOESM1_ESM.pdf]

Supplementary information for:

Internal Flow in Sessile Droplets Induced by Substrate Oscillation: Towards Enhanced Mixing and Mass Transfer in Microfluidic Systems

Tianyi Zhang, Peng Zhou, Terrence Simon and Tianhong Cui

Department of Mechanical Engineering, University of Minnesota, Minneapolis, MN 55455, USA

*Corresponding author
Email address:
cuixx006@umn.edu (Tianhong Cui)
Postal address:
111 Church Street SE, Minneapolis, MN 55455, USA

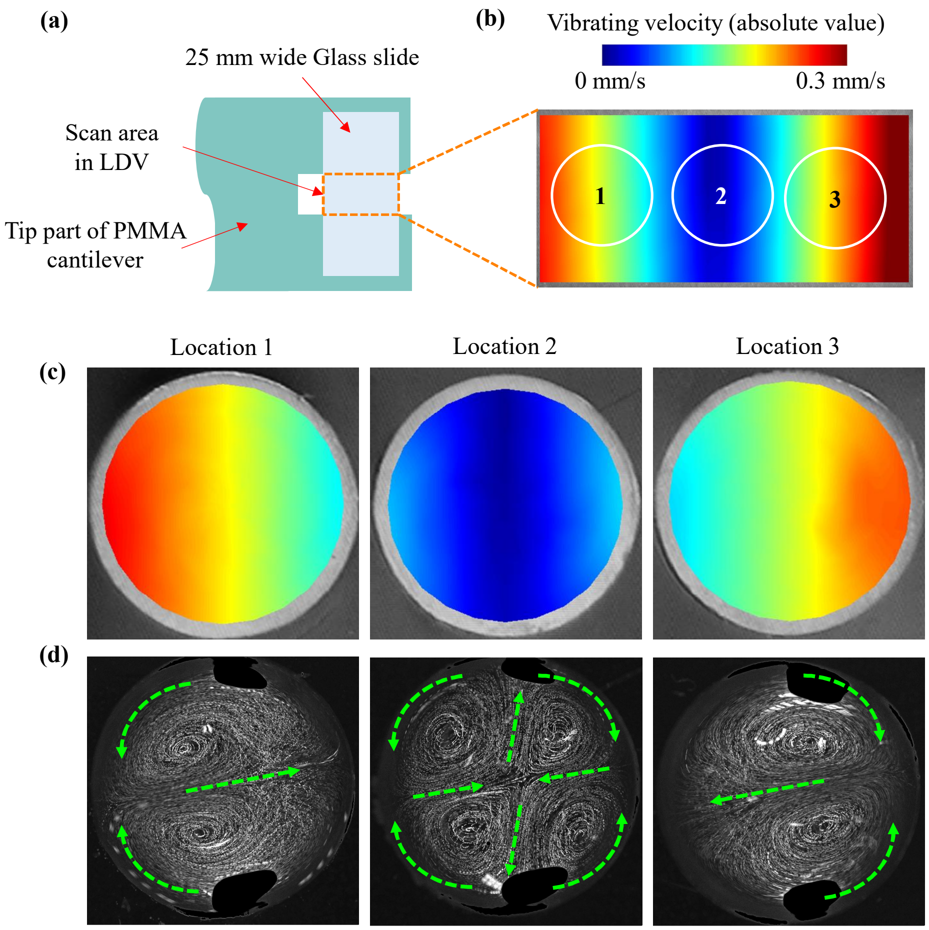

Figure S1: (a) is a schematic of the experimental setup using a 25 mm wide glass slide. (b) shows measured mode shape plots of the glass substrate. The white circles in (b) indicate the three locations where the PDMS retainer is attached. (c) are measured mode shape plots of the substrate within the contour of the three circular retainers. (d) are the flow pattern images of the sessile droplets. The flow directions are labelled with green arrows. In the above measurements, the cantilever is driven into vibration at the resonant frequency of its second-order mode, with a driving voltage of 100 mV.

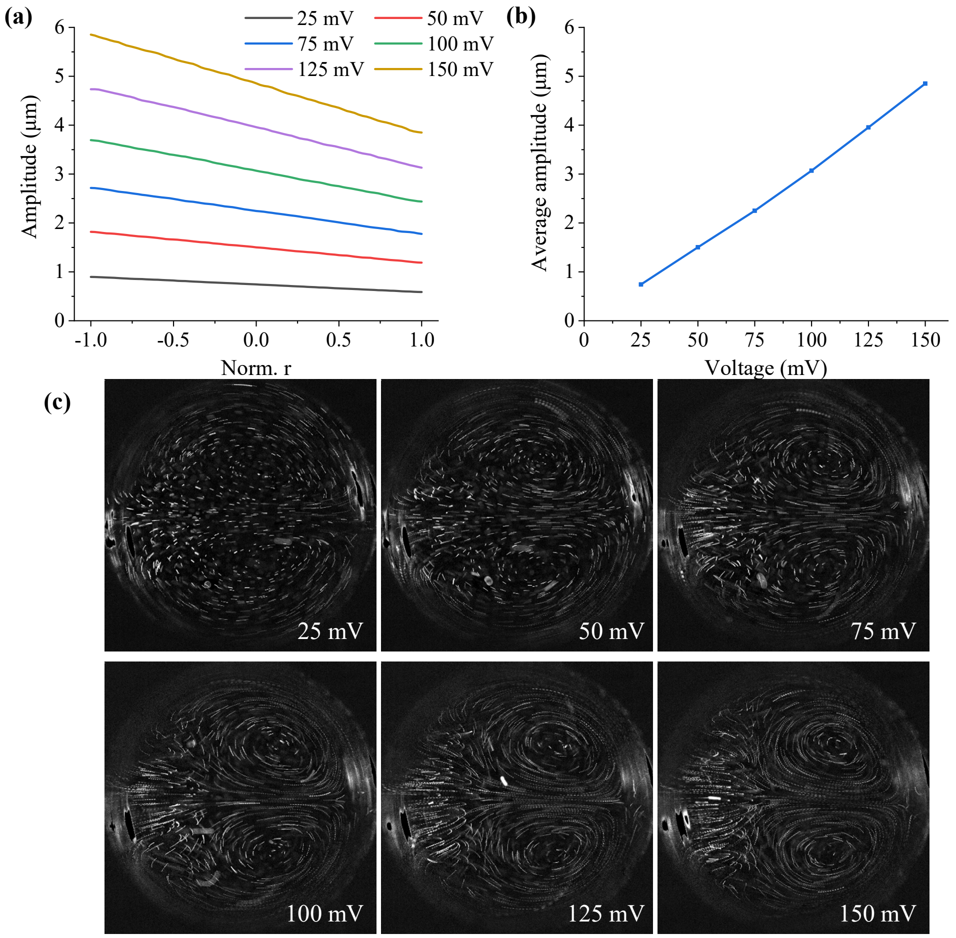

Figure S2: Plot (a) shows measured maximum vibrating amplitude distributions along the diameter of the droplet retainer under various driving voltages at a constant driving frequency of 1.15 kHz, (b) is the averaged amplitude in figure (a) as a function of the driving voltage, (c) are the flow pattern images indicated with the particle trajectories within 0.05 s under different driving voltages.

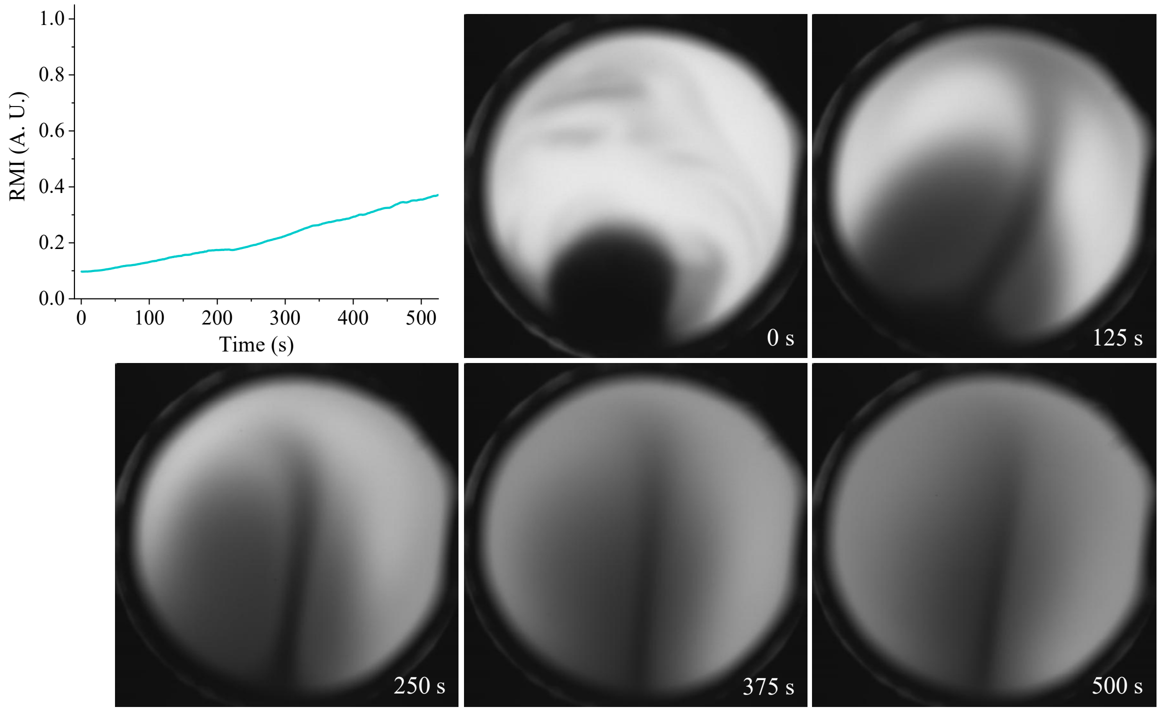

Figure S3: Relative mixing index as a function of the mixer's working time under static conditions, and optical images of sessile droplets in the retainer at different times during the mixing process. The substrate remains stationary.
